# Chronic Vaping Related Tracheomalacia (TM): A Case of Vaping Induced Altered Innate Immunity that Culminated in Severe TM

**DOI:** 10.7759/cureus.7571

**Published:** 2020-04-07

**Authors:** Abhinav Mittal, Aneeqah Baig, Rafia Zulfikar, Sunil Sharma

**Affiliations:** 1 Internal Medicine, Section of Pulmonary, Critical Care & Sleep Medicine, West Virginia University, Morgantown, USA

**Keywords:** vaping, tracheomalacia, positive airway pressure, non-invasive mechanical ventilation, electronic cigarette associated lung injury

## Abstract

Tracheomalacia (TM) is a weakness of the trachea either due to impaired cartilage integrity or atrophy of muscular elastic fibers. We present the first-ever case of chronic vaping induced altered immunological defenses that led to frequent pulmonary infections, ultimately culminating in severe TM which we successfully treated with positive airway pressure (PAP) therapy.

A 53-year-old male presented with hypoxia and pneumonia refractory to outpatient antibiotics and steroids. He underwent bronchoscopy which showed severe TM, prompting transfer to our institution. He started vaping seven years ago and noted frequent bronchitis requiring antibiotics and steroids along with 10 life-time surgeries. He underwent repeat bronchoscopy noting TM, worst 3 cm above the carina and extending 4 cm proximally. The lesion was deemed not suitable for stenting, so PAP therapy was initiated. Bronchoalveolar lavage (BAL) confirmed 40% alveolar macrophages positive for lipid in Oil-O-Red stain consistent with EVALI. He tolerated PAP therapy with significant improvement in his ground glass opacities (GGO) and TM on subsequent imaging.

TM is generally defined as >50% narrowing in the sagittal diameter. It is often further characterized into primary (congenital) or secondary (acquired) causes. Notable secondary causes include postintubation, chronic infection/bronchitis, chronic inflammation, and frequent steroid exposure -- all present in this case. Furthermore, there is existing literature that chronic inflammation due to irritants like cigarette smoke may be an important contributor to the development of TM. However, such data are lacking for EVALI.

Our patient started experiencing repeated bronchitis episodes after he started vaping, leading to chronic inflammation and frequent antibiotics/steroids. Given his additional risk factor of multiple surgeries, this case not only presents a perfect storm for TM, but also a novel manifestation of EVALI. This case, to our knowledge, is the first-ever manifestation of EVALI presenting with TM. Management with PAP therapy helped avoid major surgery.

## Introduction

Tracheomalacia (TM) is a weakness of the trachea due to either impaired cartilage integrity or atrophy of muscular elastic fibers. It has been referenced in medical literature since 1930s [1]. Modern day enhancements in imaging, bronchoscopy, and clinical recognition are helping to refine identification, causes, and treatments. Conversely, vaping is a far newer phenomenon for which bench and clinical research still lags in assessing the true spectrum and extent of electronic cigarette associated lung injury (EVALI). We present a case of chronic vaping induced altered immunological defenses that led to frequent pulmonary infections. Given his unique risk factors, we strongly believe vaping ultimately culminated in severe TM leading to hypoxic respiratory failure. Prompt initiation of positive airway pressure (PAP) therapy averted surgery. This is the first case demonstrating the manifestation of TM related to EVALI.

## Case presentation

**Figure 1:**
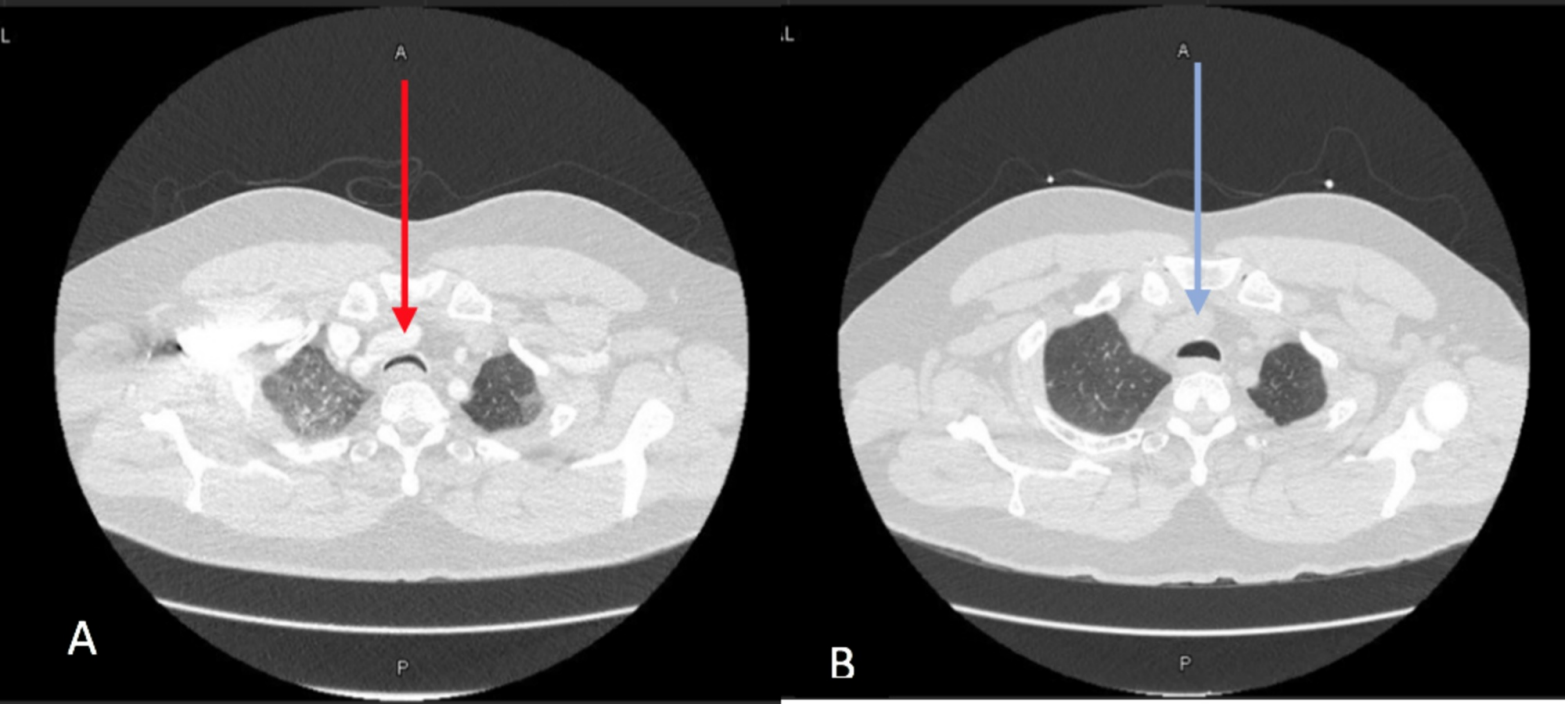
CT chest axial images showing A) severe TM (red arrow) in proximal airway along with diffuse GGO bilaterally B) showing improvement in TM (blue arrow) and GGO. TM, tracheomalacia; GGO, ground glass opacities

**Figure 2:**
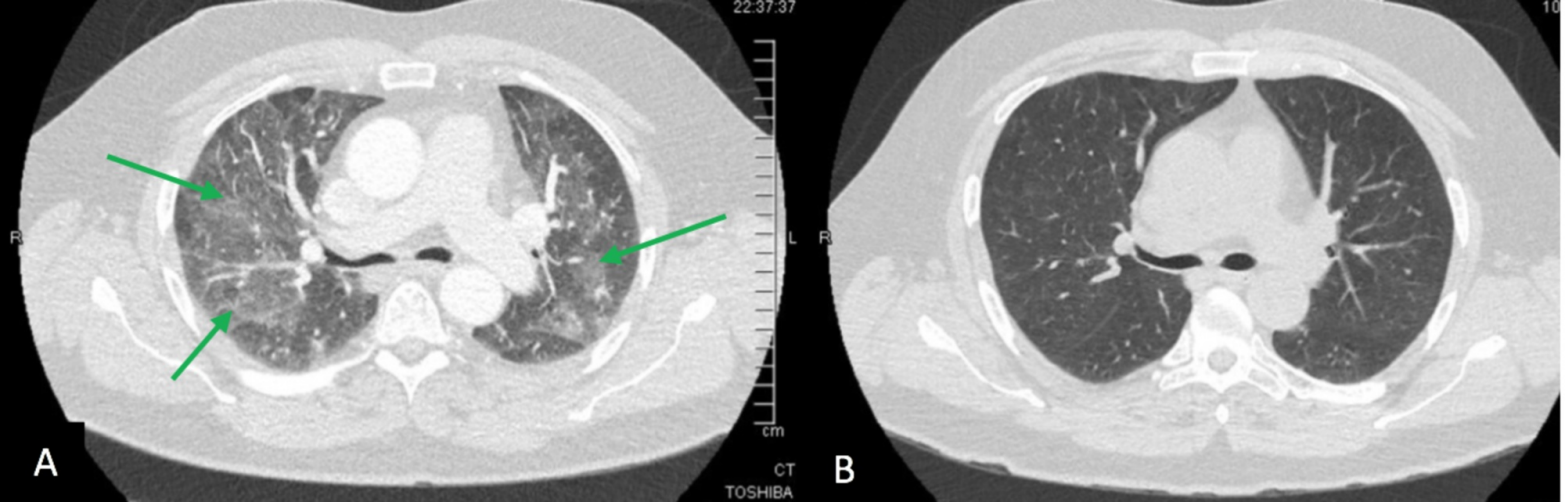
CT chest axial images showing A) severe TM in distal airway involving both mainstem bronchi and ground glass opacities (green arrows) bilaterally vs. B) improvement in TM with now patent normal caliber mainstem bronchi bilaterally and nearly resolved GGO. TM, tracheomalacia; GGO, ground glass opacities

A 53-year-old male initially presented to outside facility with hypoxia and pneumonia refractory to outpatient antibiotics and steroids. He started vaping seven years ago (never smoked cigarettes) and noted frequent bronchitis requiring antibiotics and steroids. However, the current episode was prolonged and did not respond to usual interventions. He noted severe dyspnea, wheezing, choking on minimal exertion, and episodic desaturation despite supplemental oxygen. He underwent bronchoscopy which showed severe TM, prompting transfer to our institution. He reported a lifetime total of 10 surgeries (including seven spinal surgeries) requiring procedural intubations. Interestingly, while he never had a prolonged intubation, seven of his surgeries occurred after he started vaping including five spinal revisions related to poor wound healing. No past history of gastroesophageal reflux disease (GERD) or symptoms of relapsing polychondritis were recorded. Infectious workup here showed positive rhinovirus PCR. Chest CT revealed diffuse bilateral ground glass opacities (GGO) and severe TM. He underwent repeat bronchoscopy noting TM, worst 3 cm above the carina and extending 4 cm proximally. The lesion was deemed not suitable for stenting (Figures 1A and 2A) so PAP therapy was initiated. Bronchoalveolar lavage (BAL) confirmed 40% alveolar macrophages positive for lipid in Oil-O-Red stain consistent with EVALI. He tolerated PAP therapy with significant improvement in his GGO and TM on subsequent imaging (Figures 1B and 2B).

## Discussion

Tracheomalacia is generally defined as >50% narrowing in the sagittal diameter. TM is present up to 10% of the time on computed tomography pulmonary embolism (CTPE) studies [2] and in less than 1% of all bronchoscopies [3]. Clinically, TM presents with nonspecific symptoms such as dyspnea, cough, or hemoptysis, and is often further characterized into primary (congenital) or secondary (acquired) causes [3]. Notable secondary causes include postintubation, chronic infection/bronchitis, chronic inflammation, and frequent steroid exposure-all present in this case. Furthermore, there is existing literature that chronic inflammation due to irritants such as cigarette smoke may be important contributors to the development of TM. However, such data are lacking for EVALI.

Our case highlights the pro-inflammatory state created by chronic exposure to vaping combined with potentially altered immunological defense leading to such severe respiratory failure in the setting of what is typically innocuous rhinovirus infection. In vitro studies show that vaping causes significant toxicity of alveolar macrophages, inducing a pro-inflammatory state [4]. Animal models show vaping mediated disruption of innate immunity against viral pathogens in macrophages [5]. Similarly, observational studies in humans have shown deranged innate defense proteins in airway secretions altering immune response to various pathogens, which could explain the severe respiratory compromise caused by rhinovirus infection in our patient [6]. Additionally, recent data reveal imbalance between protease-antiprotease induced by vaping leading to increased proteolytic activity. This may place patients vaping for prolonged period of time, at increased risk of developing chronic lung diseases [7]. Taken together, there is mounting evidence that vaping is not an innocuous alternative to smoking.

Our patient with no history of lung disease started experiencing repeated bronchitis episodes soon after he started vaping, leading to chronic inflammation, recurrent steroid and antibiotic exposure, and altered innate immunity leading to profound illness from rhinovirus. Given his additional risk factor of multiple surgeries, this case not only presents a perfect storm for TM, but also highlights a novel manifestation of chronic vape related injury.

## Conclusions

This patient had a unique amalgamation of risk factors that culminated in a novel manifestation of EVALI. His vaping led to recurrent infections which necessitated antibiotics and steroids. This, coupled with his many surgeries, pre-disposed him to develop TM. There is mounting evidence that vaping alters immunity and innate response, increases inflammation, and can help precipitate dramatic illness. This case, to our knowledge, is the first-ever manifestation of EVALI presenting with tracheomalacia. Proper management with PAP therapy helped resolve his hypoxic respiratory failure and avoided major surgery. Ultimately, this is a novel manifestation highlighting the danger of electronic cigarettes/vaping and adds to the differential diagnosis of clinicians that are tasked to help treat EVALI.

## References

[1] Sprague H, Ernlund C, Albright F (1933). Clinical aspects of persistent right aortic root. New Engl J Med.

[2] Hasegawa I, Boiselle P, Raptopoulos V, Hatabu H (2003). Tracheomalacia incidentally detected on CT pulmonary angiography of patients with suspected pulmonary embolism. Am J Roentgenol.

[3] Carden K, Boiselle P, Waltz D, Ernst A (2005). Tracheomalacia and tracheobronchomalacia in children and adults. An In-depth review. Chest.

[4] Scott A, Lugg S, Aldridge K (2018). Pro-inflammatory effects of e-cigarette vapour condensate on human alveolar macrophages. Thorax.

[5] Madison M, Landers C, Gu B (2019). Electronic cigarettes disrupt lung lipid homeostasis and innate immunity independent of nicotine. J Clin Investig.

[6] Reidel B, Radicioni G, Clapp P (2018). E-cigarette use causes a unique innate immune response in the lung, involving increased neutrophilic activation and altered mucin secretions. Am J Respir Crit Care Med.

[7] Ghosh A, Coakley RD, Ghio AJ (2019). Chronic E-cigarette use increases neutrophil elastase and matrix metalloprotease levels in the lung. Am J Respir Crit Care Med.

